# Three-Dimensional Shear-Wave Viscoelastographic Estimation by System Identification for Prostate Cancer Localization

**DOI:** 10.1016/j.ultrasmedbio.2025.07.025

**Published:** 2025-08-22

**Authors:** Xueting Li, Florian Delberghe, Simona Turco, David Mills, Kirk Wallace, Giuseppe Valvano, Wim Zwart, Flemming Forsberg, Daniel L. van den Kroonenberg, Hessel Wijkstra, Massimo Mischi

**Affiliations:** a Department of Electrical Engineering, Eindhoven University of Technology, Eindhoven, The Netherlands; b GE HealthCare—Technology and Innovation Center, Niskayuna, NY, USA; c Angiogenesis Analytics, ’s-Hertogenbosch, The Netherlands; d Department of Radiology, Thomas Jefferson University, Philadelphia, PA, USA; e Department of Urology, Amsterdam UMC, Amsterdam, The Netherlands

**Keywords:** Prostate cancer, Shear-wave elastography, Elasticity, Viscosity, System identification

## Abstract

**Objective:**

This study aims to estimate viscosity in vivo in the prostate using shear-wave elastography (SWE) and to evaluate its potential, alongside elasticity, for prostate cancer (PCa) localization.

**Methods:**

A cohort of 197 patients referred for radical prostatectomy at four Dutch hospitals underwent three-dimensional (3D) SWE scans. From the acquired data, voxel-based viscosity and shear-wave (SW) velocity were computed using a system identification (SI) approach. SW velocity was also calculated using the standard SW cross-correlation for comparison. Elasticity can be derived from SW velocity. Following a registration procedure, the 3D histopathological results served as ground truth. We then trained a convolutional neural network for each individual feature combined with the prostate mask. The performance was evaluated using cross-validated receiver operating characteristic (ROC) curves.

**Results:**

Both elasticity and viscosity were significantly higher in malignant prostate regions compared to benign regions (Mann–Whitney U test). The area under the ROC curve (AUC) and 95% confidence interval (CI) for voxel-wise PCa detection by viscosity from SI, SW velocity from SI, and SW velocity from cross-correlation were 0.68 (95% CI: 0.66, 0.70), 0.67 (95% CI: 0.65, 0.70), and 0.67 (95% CI: 0.65, 0.69), respectively.

**Conclusion:**

We are the first to report in-vivo voxel-based viscosity of the prostate using SWE. Combining viscosity with elasticity did not significantly improve PCa detection, likely due to system limitations, acquisition noise, and modeling simplifications. However, in 197 prostates, viscosity alone achieved an AUC comparable to that of elasticity for PCa localization, indicating its potential as a powerful adjunctive biomarker.

## Introduction

Prostate cancer (PCa) remains a major health concern in men due to its high prevalence, substantial impact on quality of life, and risks for serious complications. Globally, PCa is the second most frequent cancer and the fifth leading cause of cancer-related death [1,2]. For decades, the standard diagnostic procedure for PCa was a transrectal ultrasound-guided prostate biopsy (TRUS-GB) of up to 12 cores, performed after measuring elevated prostate-specific antigen levels or abnormal digital rectal examination (DRE) [3,4]. However, TRUS-GB carries risks of underdiagnosis and overtreatment [5,6].

Recent advances in magnetic resonance imaging (MRI) have led guidelines recommending multiparametric (mp)MRI prior to biopsy for men with suspected PCa [7,8]. Despite its benefits, mpMRI has limitations, including restrained availability, high cost, challenges with real-time imaging, and low inter-reader agreement [9]. New ultrasound modalities, such as contrast-enhanced ultrasound (CEUS), elastography, and micro-ultrasound, have also shown potential in PCa visualization [10-16]. This study focuses on acoustic radiation force (ARF)-based shear-wave elastography (SWE), a technique that has been widely used in multiple clinical applications [17-22]. Like the manual assessment of tissue hardness in DRE, SWE can evaluate tissue stiffness, but with quantitative accuracy.

Cancerous prostate tissues are typically stiffer due to excessive collagen deposition [23,24]. Studies have demonstrated that SWE holds promise for PCa detection by providing quantitative tissue elasticity values based on estimating shear-wave (SW) velocity [21,25-33]. A meta-analysis of eight studies reported pooled sensitivity and specificity for PCa detection at 0.83 (95% confidence interval (CI): 0.66, 0.92) and 0.85 (95% CI: 0.78, 0.90), respectively [25]. To date, most SWE solutions have overlooked the dispersion (frequency-dependent velocity) of the SWs, reflecting viscosity [34,35].

However, viscosity plays a vital role in the propagation and the shape of transient SWs and may also represent an additional PCa biomarker. *Ex-vivo* dynamic mechanical analysis (DMA) [28,36,37] and *in-vivo* magnetic resonance elastography (MRE) [38,39] suggest higher viscosity in cancerous prostate tissue. Yet, *in-vivo* viscosity measurements using ultrasound remain unexplored.

This study aims to provide a new dimension in estimating *in-vivo* viscosity of the prostate using SWE. We propose a system identification (SI) approach [40] for separate estimation of three-dimensional (3D) local elasticity and viscosity in 197 patients and evaluate their potential for PCa localization.

## Materials and methods

### Study population

This study was approved by the institutional review board under reference number 2020_268#B202178. A total of 715 patients were enrolled in a prospective multicenter trial conducted in the Netherlands over approximately two years (NCT04605276); the inclusion and exclusion criteria are detailed in [41]. This study is part of that trial and includes 197 patients (Table 1) referred for multiparametric ultrasound scans due to suspicion of PCa.

### Three-dimensional SWE acquisition

**Figure 1.**
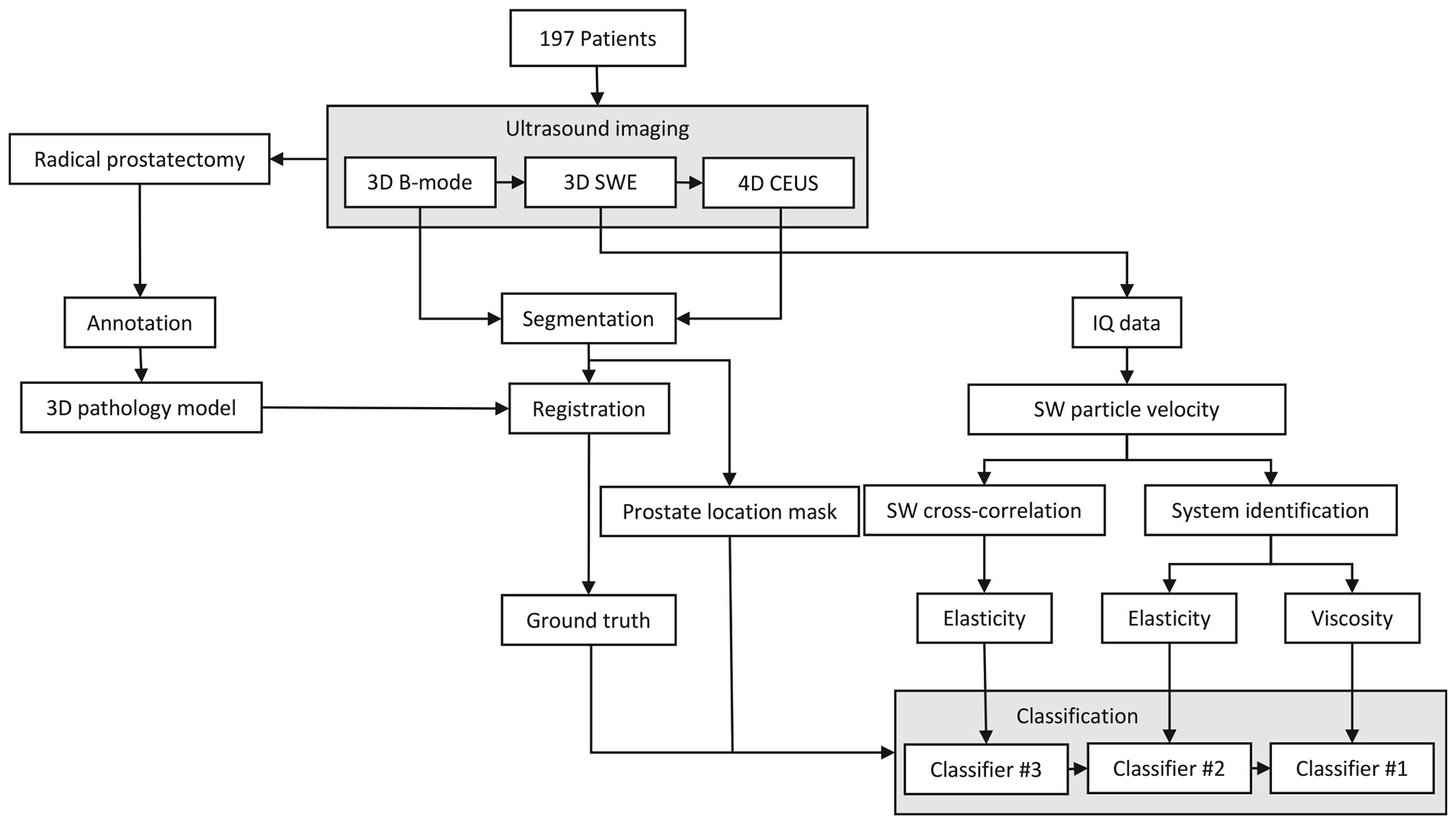
Diagram describing the acquisition and processing pipeline. 3D, three-dimensional; 4D, four-dimensional.

The study participants underwent 3D multiparametric ultrasound scans following the clinical protocol reported in [41], consisting of consecutively 3D B-mode, 3D SWE, and 4D CEUS (Fig. 1). Scans were performed with a GEHC LOGIQ^™^ E10 ultrasound scanner (GE HealthCare, Chicago, USA) with a RIC 5-9D transrectal probe. After confirming the prostate is entirely visualized, the probe was secured in a custom fixture for the duration of all acquisitions [41], maintaining consistent probe positioning and compression. All imaging parameters were fixed according to protocol for ensuring consistency and reproducibility across modalities [41].

**Figure 2.**
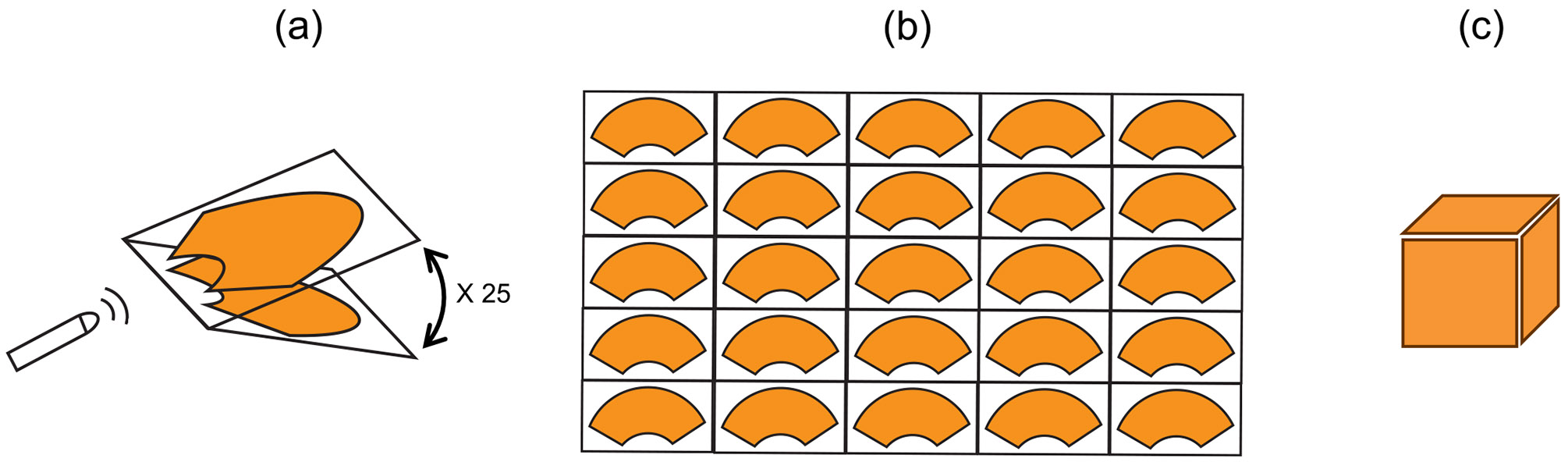
Three-dimensional (3D) shear wave elastography (SWE) acquisition and 3D viscoelastic features. (a) A motorized mechanism within the probe performs an automatic stepwise sweep, covering a 120-degree sweep from the base to the apex of the prostate in 5-degree increments. (b) A total of 25 SWE planes were acquired per patient. (c) Pixel-based elasticity and viscosity maps were computed for each SWE plane, and these 25 2D maps were then stacked in spatial order and linearly interpolated to the ground truth coordinates, resulting in 3D volumes of elasticity and viscosity.

A motorized mechanism inside the probe enables an automatic stepwise 120-degree sweep from the basal to the apical side of the prostate, with 5-degree increments, yielding a total of 25 SWE planes (Fig. 2). During acquisition, comb-push excitation (two simultaneous 3.6-MHz push beams of 0.4-ms duration in a comb-like pattern) [42,43], and time-interleaved tracking [44] were employed, and the resulting in-phase and quadrature (IQ) data was recorded for later processing.

### Three-dimensional pathology model

After surgery, the RP specimen was treated according to the protocol [41]. The specimen was fixated in formalin then sectioned into 4-mm thick slices from apex to base using the TruSlice specimen cut-up system (CellPath Ltd, Newtown, UK). Whole-mount pathology slices were then cut from prostate slices using a microtome and scanned to be annotated by pathologists blinded to clinical data [41,45]. In addition to cancerous areas, the prostate border, peripheral zone (PZ), transition zone (TZ), and visible landmarks were annotated [41]. All lesions with the International Society of Urological Pathology (ISUP) Grade Group (GG) ≥ 2 were considered malignant, following our clinical protocol [41,46].

Following the annotation, the pathology slices were overlaid on the macroscopic prostate slices and stacked to create a 3D pathology model.

### Binary ground truth

The acquired B-mode and CEUS images were segmented by two clinicians with over four years of experience, using an internally developed segmentation tool (Angiogenesis Analytics (AA), ’s-Hertogenbosch, The Netherlands). This tool allows them to visualize the ultrasound scans at a voxel resolution of 0.5 mm × 0.5 mm × 0.5 mm after scan conversion and to delineate the distinct anatomical structures. The 3D prostate pathology model was then registered onto the corresponding segmented B-mode and CEUS ultrasound images. The registration algorithm included both rigid and nonrigid transformations and compensated for *in-vivo* prostate deformation caused by pressure from the ultrasound probe, as well as *ex-vivo* deformation due to prostate excision and subsequent formalin fixation [47]. The registration tool achieved a mean target registration error of 3.5 mm (range: 0.4–5.4 mm) [48].

Following the registration procedure, voxels within registered slides were labeled as malignant or benign based on pathology annotations; for voxels between slides, interpolation techniques were applied to assign a probability of malignancy. Voxels with a probability greater than 0.5 were labeled as malignant and the rest as benign.

### Extraction of the prostate location mask

Using the segmentation tool from AA, the clinician segmented the prostate by marking points on its boundary in the B-mode image. A mesh was then computed by constraining a smooth surface to the boundary points, which continuously updated as new points were added. Finer adjustments were performed to fit the shape of the prostate better. Thus, the prostate location mask as a categorical feature was a Boolean indicating whether a voxel lies within the manually segmented prostate.

### Extraction of voxel-based elasticity and viscosity features

From the acquired IQ data, axial particle velocity time series were derived by the Loupas two-dimensional (2D) autocorrelator [49]. A third-order zero-phase Butterworth high-pass filter was applied to the velocity time series to remove low-frequency motions, with a cut-off frequency of 150 Hz. A 2D directional filter was then used to separate the left and right propagating SWs, allowing them to be processed separately [50].

To describe SW propagation dynamics, we consider the SW particle velocities at two laterally spaced pixels, ν(x,t) and ν(x+Δx,t), as the input and output of a local linear dynamic system [40]. The relationship between them can be described as

(1) $$\nu (x+\Delta x,t)=h(\Delta x,t){*}_{t}\nu (x,t)$$

where h(Δx,t) represents the impulse response that characterizes the dynamic system describing the transition from ν(x,t) to ν(x+Δx,t). Based on the Navier-Stokes equation, and assuming a Kelvin-Voigt (KV) viscoelastic model, we can derive the impulse response h(Δx,t) in the temporal frequency domain as

(2) $$H(\Delta x,\omega )=\exp\left[-\frac{j\omega |\Delta x|}{\sqrt{(\rho c_{s}^{2}+j\omega \eta )∕\rho }}\right]$$

with $\rho ,c_{s}$, and η being the mass density, SW velocity, and shear viscosity, respectively [40]. The shear elasticity (μ), or shear modulus, can be then derived from the SW velocity using μ = $\rho c_{s}^{2}$; the Young’s modulus (E) can be approximated as E ≈ 3μ.

**Figure 3.**
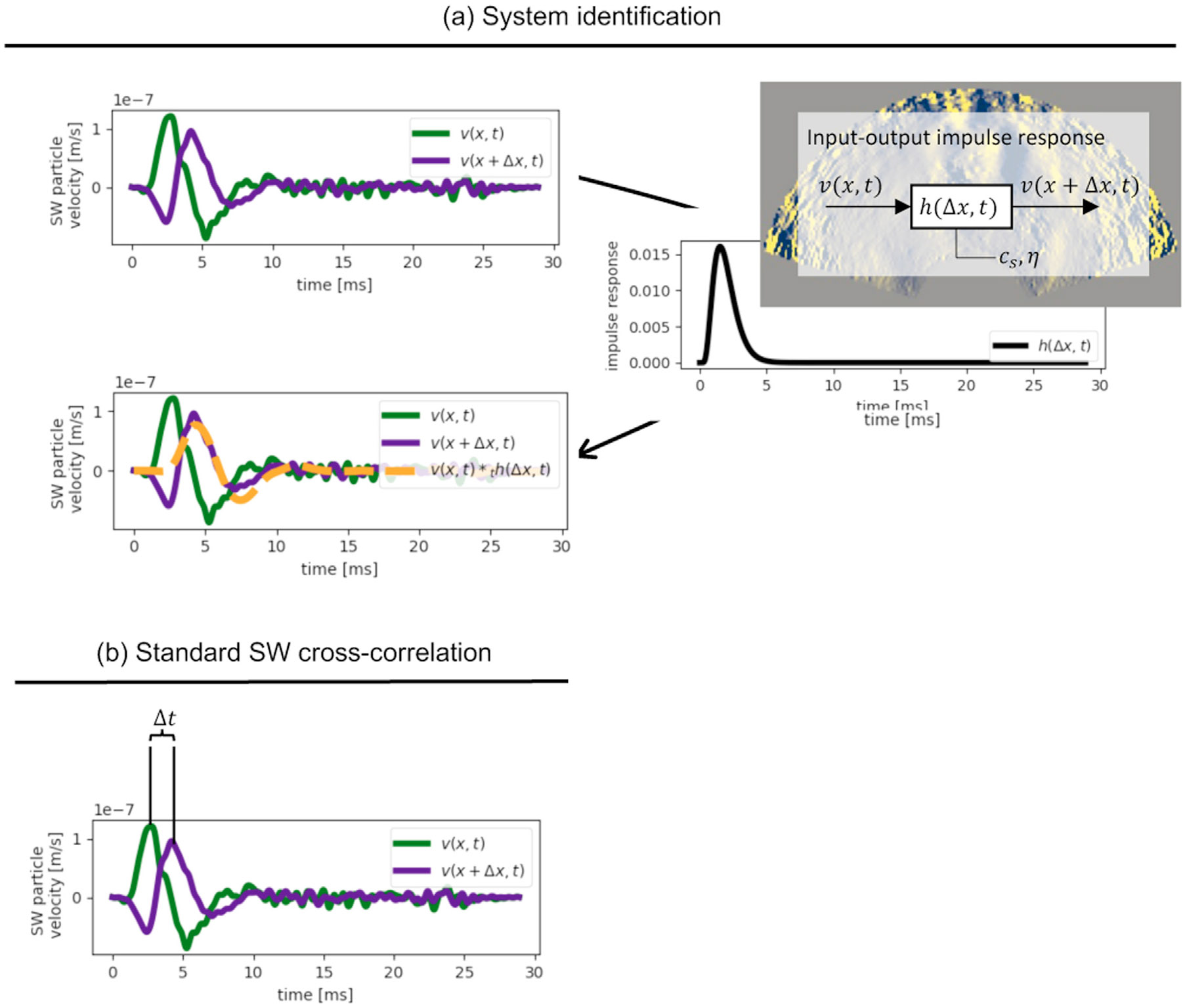
Methods for estimating local shear-wave (SW) velocity and viscosity. (a) System identification (SI) method. The SW particle velocities at two laterally spaced pixels, ν(x,t) and ν(x+Δx,t), are considered as the input and output of a local linear dynamic system. The impulse response h(Δx,t) describes the transition from ν(x,t) to ν(x+Δx,t). Using the SW particle velocity signals ν(x,t) and ν(x+Δx,t), and the model of the impulse response derived from the Navier-stocks equation, we formulated an SI problem to compute SW velocity $c_{s}$ and viscosity η. (b) Standard SW cross-correlation method. By examining the peaks of ν(x,t) and ν(x+Δx,t), the time delay between the two signals can be determined. This time delay is then used to calculate the SW velocity.

Given ν(x,t) and ν(x+Δx,t), we formulated a system identification (SI) problem (Fig. 3a). Pixel-based SW velocity and viscosity can be derived by solving the following nonlinear least-squares equation:

(3) $$\{{\hat{c}}_{s},\hat{\eta },\hat{\alpha }\}(x)={\mathrm{argmin}}_{c_{s},\eta ,\alpha }{\left\|\alpha {ℱ}^{-1}[H(\Delta x,\omega )V(x,\omega )]-\nu (x+\Delta x,t)\right\|}_{2}^{2}$$

where α is a scaling factor, ${ℱ}^{-1}$ denotes the inverse temporal Fourier transform (FT), and V(x,ω) is the temporal FT of ν(x,t). Equation (3) was numerically solved iteratively using the Nelder-Mead simplex algorithm [40].

For comparison, we also estimated the local elasticity using the standard SW cross-correlation method [51]. By examining the peak of the cross-correlation between two neighboring signals ν(x,t) and ν(x+Δx,t), the time delay between them can be determined (Fig. 3b). This time delay is then used to calculate the SW velocity and, thereby, elasticity. To evaluate signal and fitting quality, we defined two metrics: the normalized cross-correlation coefficient (NCC) between SW particle velocity signals at adjacent pixels; and the coefficient of determination (*R*^2^) from the SI fitting process.

With the aim of achieving the maximum spatial resolution by minimizing the distance Δx, this was fixed to 6 pixels (4.5 mm). This allows for resolving the SW time shift even for maximum velocities of 10 m/s (Young’s modulus of 300 kPa), which is well above the highest reported values for prostate tissue [15,52].

Pixel-based elasticity and viscosity maps were computed for each SWE plane. These 25 2D maps were then stacked in spatial order and linearly interpolated to the ground truth coordinates, enabling the extraction of voxel-based viscoelastic features (Fig. 2).

### Classification procedure

Classifiers were then trained for the combination of prostate location mask with SI-derived viscosity, SI-derived SW velocity and SW cross-correlation-derived SW velocity. To assess whether combining SI-derived features improves performance, we also trained a classifier using both SI-derived features and the prostate location mask. We then repeated the training with all feature combinations using voxels from either the PZ or TZ, to evaluate the zone-specific performance.

To account for the spatial distributions of the features, a small (2k trainable parameters) convolutional neural network (CNN) was trained [53,54] and validated using patient-wise five-fold cross validation (repeated three times). The network was trained using the Adam optimizer [55] with a learning rate of 0.01, for 100 epochs. Each epoch consisted of 50,000 samples with a batch size of 128. The binary cross-entropy loss function was used for optimization, without additional regularization on the weights. To minimize bias across folds, patients were first subdivided into four groups according to the severity of PCa and the groups were evenly distributed across folds. During training, each batch was generated with a 50% prevalence rate to balance the number of voxels across the two classes. The average receiver operating characteristic (ROC) curves were evaluated over each validation set. The area under the curve (AUC) and the 95% CI were computed.

## Results

The demographics of the study sample and the process diagram are provided in Table 1 and Figure 1, respectively. In the following results, we use directly the SW velocity cs instead of elasticity μ. Elasticity can be derived using μ = $\rho c_{s}^{2}$, with mass density ρ assumed to be 1000 kg/m^3^.

### Voxel-based SW velocity and viscosity features

**Figure 4.**
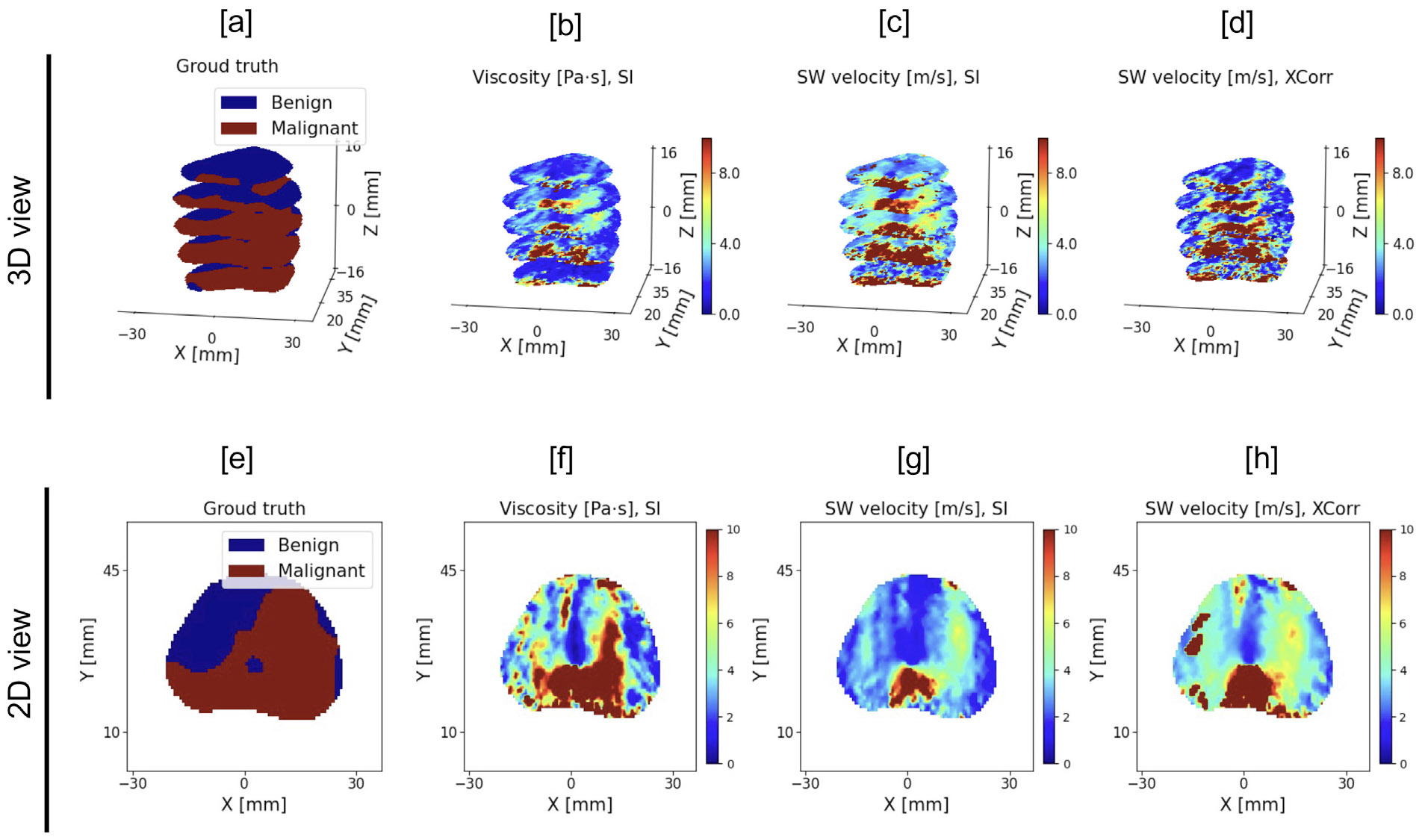
Ground truth, viscosity, and shear-wave (SW) velocity maps of one representative patient. The top row shows the three-dimensional (3D) view, with five planes displayed from the 3D volumes for clearer visualization; the bottom row presents the two-dimensional (2D) view, displaying the middle plane. (a) 3D ground truth. (b) 3D viscosity derived from the system identification (SI) method. (c) 3D SW velocity derived from the SI method. (d) 3D SW velocity derived from the standard cross-correlation (XCorr) method. (e) 2D ground truth. (f) 2D viscosity derived from the SI method. (g) 2D SW velocity derived from the SI method. (h) 2D SW velocity derived from the XCorr method.

Figure 4 presents both 3D and 2D visualizations of SW velocity and viscosity features, alongside the ground truth for a representative patient. For this patient, SI-derived viscosity and SW velocity values from both methods were higher in malignant regions compared to benign ones, while the contrast between benign and malignant regions was less pronounced in cross-correlation-derived SW velocity than in the SI-derived features. For all patients, Table 2 lists the mean and standard deviation of viscoelastic features in malignant and benign regions, respectively. The *p*-values calculated using the Mann–Whitney U test (MWU) and the Cliff’s Delta effect size [56] are reported Table 2.

**Figure 5.**
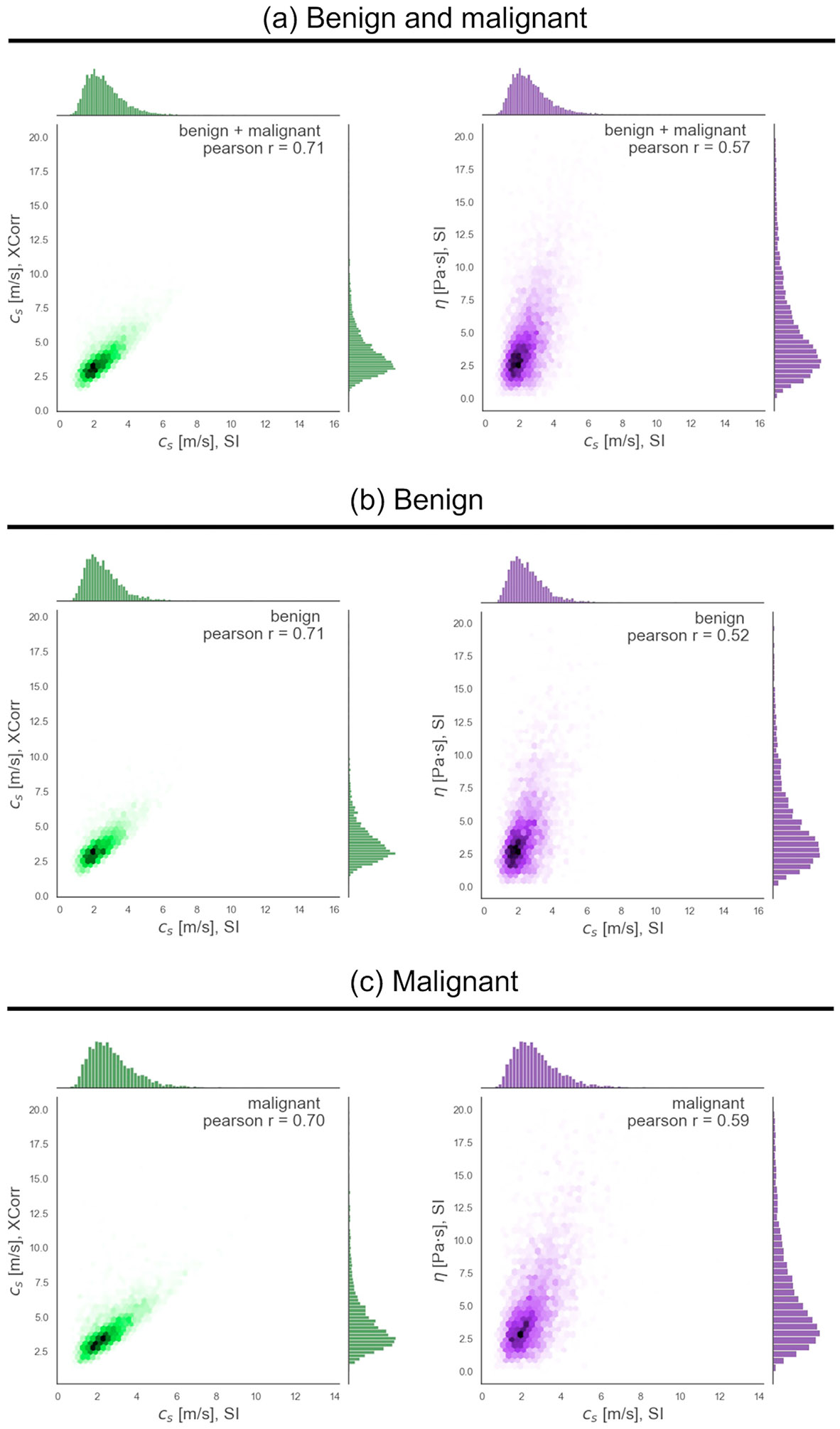
Hexagonal binned plots of shear-wave (SW) velocities cs obtained from the system identification (SI) method and the SW cross-correlation (XCorr) method, as well as between viscosity η and SW velocity derived from the SI. Correlations are shown for all voxels across the entire patient cohort in three scenarios: (a) including both classes, (b) the benign class alone, and (c) malignant class alone.

Besides, we examined the correlation between voxel-based SW velocities obtained using both methods of all patients, and between viscosity and SW velocity derived from SI. To balance the dataset, we randomly down-sampled voxels from the majority class (benign) to match the number of voxels in the minority class (malignant). Figure 5 displays the hexagonal binned plots of these data, showing correlations across all voxels in three scenarios: including both classes, the benign class, and the malignant class.

As observed from Figure 5, the SI-derived SW velocity values strongly align with the cross-correlation-derived SW velocity values, consistently yielding a Pearson correlation coefficient (PCC) of 0.7 across all three scenarios. The correlation between SW velocity and viscosity from SI tends to be strong, with a PCC of 0.57. Notably, this correlation is lower for the benign class (0.52) while higher for the malignant class (0.59).

### Classification performance

**Figure 6.**
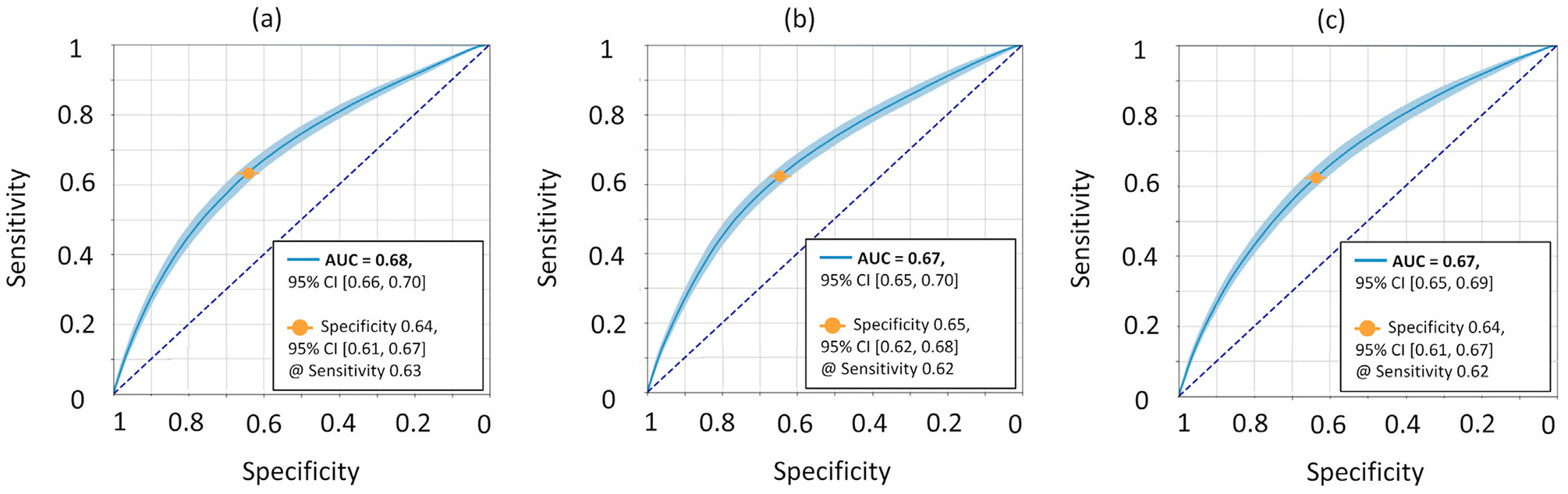
Receiver operating characteristic curves for: (a) prostate location mask combined with viscosity feature derived from the system identification (SI) method, (b) prostate location mask combined with SW velocity feature derived from the SI method, and (c) prostate location mask combined with SW velocity feature derived from the standard shear-wave cross-correlation method. AUC: area under the curve; CI, confidence interval.

Figure 6 illustrates the ROC curves when combining the prostate location mask with one of the SWE-derived features. In addition, Table 3 lists the AUC values and 95% CI. The AUC value and 95% CI of using both SI-derived features are also included in Table 3. Table 4 shows the results for classifiers trained on voxels from the PZ and TZ, respectively.

As shown in Figure 6 and Table 3, combining the prostate location mask with SW velocity features from both methods achieved comparable results, with an AUC of 0.67. The use of viscosity resulted in a slightly higher AUC with an increase of 1.49%. However, Table 3 shows that combing both SI-derived features did not lead to a statistically significant improvement in the AUC values compared to using SI-derived SW velocity (*p*-value = 0.803, MWU test). A comparison of AUC values and their CI in Table 4 reveals a significant difference in performance between the PZ and the TZ for the analyzed features (*p*-value < 0.001, MWU test), with better performance in the PZ than the TZ.

## Discussion

In this study, we calculated 3D *in-vivo* voxel-based SW velocity and viscosity features from 197 patients and evaluated their potential for PCa localization using CNN.

*Ex-vivo* studies using sonoelastography and SW elasticity imaging (SWEI) reported shear modulus from 1.8 to 10.8 kPa in normal prostate tissue and 10 to 20 kPa in cancerous tissues [28,37,57,58]. Mitri, et al. examined multiple locations in three excised noncancerous human prostates using shear-wave dispersion ultrasound vibrometry (SDUV), reporting elasticity values between 1.3 and 12.8 kPa [57]. A more recent publication in 2021 performed 3D SWEI on 36 patients and reported the mean SW velocities of 4.8 m/s, 5.3 m/s, and 6.0 m/s for the PZ, CZ, and PCa lesions, respectively [15]. The AUC for the mean SW velocity in separating malignant from benign lesions was 0.84 [15]. Additionally, an AUC of 0.67 for pixel-based Young’s modulus and 0.66 ± 0.12 for region-based Young’s modulus were reported in [32,33].

In our study, the mean elasticity value obtained from SI is closer to the reported range in [28,37,57,58] compared to the value derived from cross-correlation. The cross-correlation approach overlooks the impact of viscosity, leading to an overestimation of elasticity values [59]. Our AUC results for SW velocity are comparable to those reported regarding Young’s modulus in [32,33]; however, one study used 2D SWE, while the other employed supersonic shear imaging (SSI) with biopsy outcomes as the ground truth.

The study in [15] is closely related to ours, as both demonstrate the use of 3D SWE to differentiate malignant from benign prostate tissue, using registered whole-mount histopathology as the ground truth. Both studies employed similar signal processing techniques, including the Loupas autocorrelator, Butterworth filtering, and directional filtering. However, our study included five times as many patients. The imaging systems differed: [15] used an external rotation stage for 3D acquisition, while we used a fixed probe with internal volumetric sweeping. Regarding SW generation and tracking, [15] employed denser ARF pushes at multiple depths and higher resolution tracking, likely yielding broader SW bandwidth and higher signal-to-noise ratio (SNR). Clinically significant PCa was defined differently; [15] included only ISUP GG > 2, whereas we also included GG = 2. SW velocity estimation also varied: [15] used 2D cross-correlation, and we computed velocity laterally. They applied a region-based analysis, while we performed a voxel-based CNN classification. These differences likely contributed to the lower SW velocity values and AUC in our results.

To date, only a few studies have addressed the estimation of viscosity in the prostate. Using MRE at 3T, Li et al. reported mean viscosity values of 6.56 ± 0.99 Pa·s for PCa lesions in 28 men [39]. In a separate study using multifrequency MRE [38], viscosity was not directly measured but instead represented by the loss angle, which is associated with viscosity-related fluidity. *Ex-vivo* DMA were used to estimate viscosity employing a quasi-linear viscoelastic model [36] and a KV fractional derivative viscoelastic model [28,37]. In an *ex-vivo* study using the KV viscoelastic model, Mitri et al. reported viscosity values ranging from 1.10 to 6.82 Pa·s [57] in three healthy prostates using SDUV. To our knowledge, no AUC values have been reported so far for viscosity.

In our study, the mean *in-vivo* viscosity value (5.49 Pa·s) is close to *in-vivo* MRE results for PCa lesions (6.56 Pa·s) [39]. However, our results are not directly comparable to DMA, as we used a different viscoelastic model to fit viscosity. The viscosity values derived from benign voxels in our study fall within the range reported in [57], an *ex-vivo* study using the KV viscoelastic model on noncancerous prostate tissue. While *ex-vivo* and *in-vivo* conditions differ due to the *ex-vivo* lack of perfusion, lower temperature, and absence of surrounding anatomical constraints, this comparison serves only as a contextual reference and not as a direct validation.

In line with above mentioned literature, our study observed that both SW velocity/elasticity and viscosity are higher in malignant prostate lesions compared to benign ones, as shown in Table 2. Although the MWU test indicated a statistically significant difference between malignant and benign groups, the effect size, as measured by Cliff’s Delta, was negligible. This suggests that, despite a consistent trend of higher values in malignant lesions, the magnitude of the difference may have limited practical or clinical relevance. The small effect size is attributed to overlap between the two groups, which may result from measurement limitations, the complexity of prostate tissue, and the inherent variability of *in-vivo* data driven by multiple technical and physiological factors. Higher viscosity observed in malignant prostate lesions may result from extensive changes in proteoglycans (PGs) within the extracellular matrix of the stroma [60]. PGs are regarded as the main viscous constituents, embedding the collagen fibers and creating a lubricating effect [35].

Additionally, we noticed a significant difference in AUC values when using features from the PZ and the TZ, as shown in Table 4. While PCa is more prevalent in the PZ, we observed that both NCC and *R*^2^ values were lower in the TZ, indicating reduced signal quality. The median *R*^2^ values across all voxels and patients were 0.45 (interquartile range (IQR): 0.13–0.62) in the PZ and 0.15 (IQR: 0.08–0.44) in the TZ, while the corresponding NCC values were 0.47 (IQR: 0.30–0.67) and 0.31 (IQR: 0.27–0.44), respectively. This degradation is likely due to the TZ being located further away from the probe, resulting in stronger signal attenuation. Moreover, shadowing from the urethra in the TZ, and biological differences between the PZ and TZ may also contribute to the observed difference in performance. When features from both zones were combined, as seen in Table 3, the AUC increased. This improvement can be ascribed to the ability of the CNN model to learn the spatial information reflecting zones from the features, enhancing overall performance.

Our findings strengthen the evidence for the clinical utility of SWE in PCa detection and further demonstrate the feasibility of our SI framework for *in-vivo* voxel-based viscoelasticity mapping. The elasticity values closely matched those from the standard cross-correlation method, confirming the reliability of our approach. Given its generalizability, the SI framework can readily be extended to other cancer applications.

The current study has several limitations. First, we observed that the SWs attenuated quickly in the region of interest (ROI) with the current push duration. While a longer push duration could be applied to deliver higher energy, it would also produce a compression of the SW spectral content [61], which affects the frequency-dependent viscosity measurements. Therefore, finding the optimal duration requires balancing the delivered energy and the resulting spectra while meeting FDA regulations.

Second, in SWE acquisition, comb-push was used to interrogate a larger tissue region in a single transmit event. However, ARF was focused on a single depth, resulting in strong attenuation in the far-field, where the TZ is located. Time-interleaved tracking with a focused beam was employed so that measuring the tissue response in the entire ROI only requires a single push event, while the missing data points in time were approximately recovered by interpolation, achieving a virtual pulse repetition frequency of 3.25 kHz. Moreover, only 25 planes were acquired per patient, and the missing out-of-plane information was linearly interpolated. These led to low IQ data resolution, suboptimal feature mapping, ultimately impacting the CNN performance. To improve this, SSI with ultrafast tracking at up to 6 kHz could be used. SSI successively transmits multiple ARF pulses at different depths along the same axial line at a very high rate, enhancing the SW signal [62]. More planes should be acquired per patient to reduce the gap between the planes. However, these require hardware upgrades and an extended acquisition time.

Third, combining viscosity and elasticity values did not improve the CNN classification performance, with the AUC (0.68) remaining comparable to that achieved using viscosity (0.68) or elasticity (0.67) alone. This aligns with findings from [63], where adding KV viscosity to elasticity did not improve differentiation in liver fibrosis staging. Several factors likely contributed to this plateau. The SI approach is based on the linearized Navier-Stokes equation, which describes SW propagation in an isotropic, homogeneous, nearly incompressible medium [20]. It also assumes a single-relaxation KV viscoelastic model. However, prostate tissue exhibits more complex mechanical properties, and these assumptions may oversimplify the true viscoelastic behavior, particularly affecting the accuracy of viscosity estimates. In addition, viscosity estimation depends on the available SW bandwidth and SNR. Indeed, the fixed push focus and lack of ultrafast tracking can result in reduced SNR. These factors, combined with the challenges of accurately identifying system parameters from noisy and narrow-bandwidth data, can further limit the accuracy of the viscosity measurements.

Despite these limitations, viscosity alone yielded an AUC comparable to that of elasticity, suggesting that it captures pathological information and remains a promising biomarker. In fact, viscosity showed a moderate correlation with elasticity (PCC = 0.57), indicating that the two parameters reflect distinct mechanical properties. Future work should incorporate more advanced models of SW propagation and viscoelastic behavior, along with improved acquisition to achieve broader bandwidth and higher SNR in SW signals. Additionally, exploring other mechanical properties, such as nonlinearity and anisotropy, may further enhance SWE-based diagnostics and better reflect pathological changes in soft tissue.

While this study used a binary ground truth to detect clinically significant PCa, prior 2D SWE studies have shown that prostate stiffness increases with GG [64,65]. In contrast, [15], using 3D SWE, found no significant differences in region-based SW velocity across GG. Our voxel-level analysis allows a more localized evaluation of this relationship. Future work could explore whether correlations between reconstructed SWE features and tumor aggressiveness exist using a multi-class ground truth.

As a final note, this study focuses on the individual performance of SWE. However, previous research suggests that combining CEUS with SWE can improve PCa detection by integrating complementary information on tumor microvasculature changes due to angiogenesis and tissue viscoelastic properties [32,33]. Similar combinations (without viscosity), often referred to as multiparametric ultrasound, have already shown increased positive predictive value and decreased false positive rates in PCa detection [66]. With the availability of both modalities in our dataset, a logical next step will be to integrate SWE-derived viscoelastic features with perfusion biomarkers from quantitative CEUS analysis to achieve more accurate and robust localization of PCa. Additionally, another multiparametric ultrasound study combining ARF impulse (ARFI) imaging displacement, SW velocity, B-mode brightness, and midband fit derived from quantitative ultrasound analysis of B-mode IQ data demonstrated improved lesion visibility in PCa [67]. Incorporating echogenicity information from B-mode imaging, which reflects tissue scattering properties, together with vascular (CEUS) and mechanical parameters (SWE/ARFI) may further improve PCa detection by leveraging complementary biomarkers.

## Conclusions

In conclusion, we are the first to quantify the viscosity of the prostate *in vivo*, using 3D SWE. Our findings are consistent with prior literature and further support the clinical utility of SWE in PCa detection. Both viscosity and elasticity values are higher in malignant prostate lesions compared to benign ones. However, combining viscosity and elasticity did not significantly enhance PCa detection, likely due to system limitations, acquisition noise, and oversimplified assumptions in SW propagation and KV viscoelastic modeling. Nonetheless, viscosity alone achieved an AUC of 0.68 (95% CI: 0.66, 0.70), which is comparable to that of elasticity, indicating its potential as a powerful adjunctive biomarker. Future research should focus on improving hardware and algorithms, as well as exploring more advanced biomechanical modeling approaches.

## Figures and Tables

**Table 1:** Demographics of the patient population included in this study

Number of patients	197
• Amsterdam University Medical Center (Amsterdam, The Netherlands)	43
	129
• Anthony van Leeuwenhoek Ziekenhuis (Amsterdam, The Netherlands)	22
	3
• Andros Clinics Arnhem (Arnhem, The Netherlands)	
• Canisius Wilhelmina Hospital (Nijmegen, The Netherlands)	
Mean age ± standard deviation	68.40 ± 5.93
Number of men vs woman	197 men
ISUP GG count	
• Benign, n (%)	40 (20.30%)
• 1, n (%)	3 (1.52%)
• 2, n (%)	52 (26.40%)
• 3, n (%)	62 (31.47%)
• 4, n (%)	28 (14.21%)
• 5, n (%)	12 (6.09%)

ISUP, International Society of Urological Pathology; GG, grade groups.

**Table 2:** Mean and standard deviation of features in malignant and benign regions across all patients

Features	Malignant (mean ± standard deviation)	Benign (mean ± standard deviation)	*p*-values	Cliff’s Delta
Viscosity (SI)	5.49 ± 3.80 Pa·s	4.67 ± 3.40 Pa·s	0.018	0.066
SW velocity (SI)	2.79 ± 1.27 m/s	2.59 ± 1.13 m/s	0.032	0.060
Elasticity (SI)	9.41 ± 10.80 kPa	7.91 ± 8.81 kPa	0.032	0.060
SW velocity (XCorr)	4.45 ± 2.12 m/s	3.95 ± 1.74 m/s	0.001	0.091
Elasticity (XCorr)	24.25 ± 30.93 kPa	18.67 ± 23.18 kPa	0.001	0.091

Elasticity is derived using the relation $\mu =\rho c_{s}^{2}$ where ρ represents mass density (assumed to be 1000 kg/m3) and $c_{s}$ the shear-wave (SW) velocity. For each feature, the *p*-values (from the Mann–Whitney U test) and Cliff’s Delta effect size are reported.

SI, system identification; XCorr, cross-correlation.

**Table 3:** The area under the curve (AUC) and corresponding 95% confidence interval (CI) for different feature sets

Features	AUC (95% CI)
Viscosity (SI) + prostate location mask	0.68 (0.66, 0.70)
SW velocity (SI) + prostate location mask	0.67 (0.65, 0.70)
SW velocity (XCorr) + prostate location mask	0.67 (0.65, 0.69)
Viscosity (SI) + SW velocity (SI) + prostate location mask	0.68 (0.66, 0.70)

**Table 4:** The area under the curve (AUC) and corresponding 95% confidence interval (CI) for different features sets from the peripheral zone (PZ) and the transition zone (TZ), respectively. *p*-values were computed using the Mann–Whitney U test

Features	AUC (95% CI), PZ	AUC (95% CI), TZ	*p*-values
Viscosity (SI) + prostate location mask	0.60 (0.58, 0.61)	0.50 (0.48, 0.52)	< 0.001
SW velocity (SI) + prostate location mask	0.62 (0.60, 0.63)	0.50 (0.48, 0.51)	< 0.001
SW velocity (XCorr) + prostate location mask	0.63 (0.62, 0.64)	0.50 (0.48, 0.52)	< 0.001
Viscosity (SI) + SW velocity (SI) + prostate location mask	0.62 (0.61, 0.64)	0.51 (0.49, 0.53)	< 0.001

## Data Availability

The data supporting the findings of this study are confidential and are not publicly available. However, they may be made available upon reasonable request to the corresponding author, subject to confidentiality agreements and approval.
